# The Hospital Crisis: The Dangers of Unbelief

**Published:** 1911-12-09

**Authors:** 


### The Hospital

The Workers' Newspaper of Administrative Medicine and Institutional Life, Administration, National Insurance and Health.

No. 1326, Vol. LI. SATURDAY, DECEMBER 9TH, 1911. PRICE ONE PENNY.

### THE HOSPITAL CRISIS: THE DANGERS OF UNBELIEF.

The meeting of the British Hospitals Association on December 1 is fully reported in another column. It was the most representative meeting of voluntary hospital managers ever held, and there was a sincere desire on the part of those present to get to business and do something to uphold and safeguard the voluntary system of hospitals. There was good reason to hope that at the eleventh hour Mr. Lloyd George would realise the injustices of the Bill, the revolution which the Bill may create in our present hospital system, and the cruelties with which it threatens the workmen of the United Kingdom who at present enjoy free hospital treatment at the voluntary hospitals.

It was not possible to promulgate an organised plan of action for the voluntary hospitals all over the country until the Government's decision was known. To the surprise and regret of all who are familiar with the facts, the Government has not seen fit to provide in the Bill for the hospital care which the workmen throughout the United Kingdom must have when seriously ill. Yet Mr. Lloyd George told the deputation of the British Hospitals Association (see The Hospital for July 29, 1911, page 451) that insured persons would not be eligible for free relief at the hospitals, so that the lot of the British workman after the passing of this Bill may prove to be more cruel than that of any other workman in any other country in the world. Not only is the insured made ineligible for free hospital care, but, by being guaranteed adequate medical treatment in virtue of the toll paid by himself, his employer, and the State, the revenue of the voluntary hospitals is seriously threatened, and may be materially reduced, by this legislation

The voluntary hospitals and the workmen throughout the United Kingdom, by the passing of this Bill, will find themselves at the parting of the ways. The great danger of disaster arises from the firm unbelief in the dangers by which, in truth, both are threatened through this Bill. This unbelief dominates certain past and present managers of London hospitals to such an extent that they have advocated the policy of "do nothing but wait and see." We publish in another column a letter from Mr. Sydney Holland, who, with the easy-going public and some newspaper editors, cannot persuade himself that in fact the insured will ever be denied the continuance of the free treatment they at present enjoy at the voluntary hospitals. Mr. Lyttelton and Mr. Lloyd George have replied, in advance, to Mr. Holland. On the other hand, the great body of hospital opinion holds to the view, which we share, that the Bill threatens the whole of our voluntary system; that it disqualifies the insured, who are guaranteed by the State adequate medical treatment, from obtaining free in-patient benefit at the hospitals; that, in any case, the number of beds at present available in these hospitals must be diminished by the serious reduction of their present revenue; and that such reduction will be infinitely greater in the case of any voluntary hospital which continues, without question, to give free in and out patient treatment to the insured. This latter policy assumes that the workmen will pay their insurance money and gladly accept as charity hospital treatment, when they need it, if they can obtain it, despite the fact that the promoters of the Bill have emphatically declared that it gives to the workmen of the United Kingdom adequate medical treatment, free from the taint of charity.

Each voluntary hospital has therefore to make its choice. Will it let things drift and take the risk of losing much or all of its charitable revenues, only resolving to close beds to the extent by which those revenues are diminished from year to year; or are managers, as men of business, to safeguard their institutions against the risks to which Mr. Lloyd George and the Government have subjected them? Clearly prudence demands that every hospital manager worthy of the name, as a custodian of the means whereby the breadwinners of this country are provided with adequate hospital treatment, must be up and doing, and he cannot take upon himself the responsibility and disaster which under a policy of "wait and see" would make him and his colleagues entirely responsible for the evil consequences which almost certainly will result to the workmen of the United Kingdom.

It is perfectly plain, whatever certain London hospital managers may think and do, that the representatives of the two hundred hospitals present on December 1 will not be content to drift, but will certainly take prompt action, by organisation, to remove all risks of injury to the voluntary hospitals and to the workmen throughout the country. They will also combine to dispel the present unbelief, wherever they find it. They will further place themselves at once in communication with the leaders of the working classes in their own districts, and take counsel together as to what steps they shall adopt, in co-operation, to secure, that the grievous blot upon the Bill of having recruited upwards of 400,000 in-patients, without providing one single hospital bed for their reception, shall be removed. The present is not a time for hesitation or delay. Those of us who have devoted many years of our lives to the extension of the voluntary system and its tender care and regard for the individual patient, must be up and doing. Organise, organise, organise is the motto, and how to organise and suggestions of the best way to do it will be placed in the hands of the representatives of the voluntary hospitals immediately.

We have therefore made our present issue the Defence Number. We have decided to open our columns to all sides of hospital opinion, are publishing the facts upon which that opinion must be formed, and are prepared to extend co-operation to every hospital worker throughout the United Kingdom. The present is not a time to be careless or idle or indifferent, much less to cultivate unbelief in the face of the writing on the wall. We are certain that the views we have advocated, based as they are upon provable facts, represent the overwhelming majority of voluntary hospital opinion in regard to this Bill and its consequences. Let us then join hands and never rest until we have placed the voluntary hospital system upon a sound basis, by obtaining the amendment of the Bill and the insertion of hospital benefit.

